# A novel CNN architecture for accurate early detection and classification of Alzheimer’s disease using MRI data

**DOI:** 10.1038/s41598-024-53733-6

**Published:** 2024-02-12

**Authors:** A. M. El-Assy, Hanan M. Amer, H. M. Ibrahim, M. A. Mohamed

**Affiliations:** 1 Electronics and Communications Engineering Department, Faculty of Engineering, Mansoura University, Mansoura, Egypt; 2 Communication and Electronics Engineering Department, Nile Higher Institute for Engineering and Technology-IEEE Com Society Member, Mansoura, Egypt

**Keywords:** Alzheimer’s disease, Convolutional neural network, Deep learning, Intelligent systems, Explain ability, Biotechnology, Neuroscience, Health care, Medical research, Engineering

## Abstract

Alzheimer’s disease (AD) is a debilitating neurodegenerative disorder that requires accurate diagnosis for effective management and treatment. In this article, we propose an architecture for a convolutional neural network (CNN) that utilizes magnetic resonance imaging (MRI) data from the Alzheimer’s disease Neuroimaging Initiative (ADNI) dataset to categorize AD. The network employs two separate CNN models, each with distinct filter sizes and pooling layers, which are concatenated in a classification layer. The multi-class problem is addressed across three, four, and five categories. The proposed CNN architecture achieves exceptional accuracies of 99.43%, 99.57%, and 99.13%, respectively. These high accuracies demonstrate the efficacy of the network in capturing and discerning relevant features from MRI images, enabling precise classification of AD subtypes and stages. The network architecture leverages the hierarchical nature of convolutional layers, pooling layers, and fully connected layers to extract both local and global patterns from the data, facilitating accurate discrimination between different AD categories. Accurate classification of AD carries significant clinical implications, including early detection, personalized treatment planning, disease monitoring, and prognostic assessment. The reported accuracy underscores the potential of the proposed CNN architecture to assist medical professionals and researchers in making precise and informed judgments regarding AD patients.

## Introduction

An ailment of the brain called Alzheimer’s disease (AD) has become increasingly common over time and now ranks as the fourth leading cause of mortality in industrialized nations. Memory loss and cognitive impairment represent the most common signs of AD, stemming from the death and destruction of memory-related nerve cells in the brain^1^. Between normal brain function and AD lies a condition known as mild cognitive impairment (MCI)^2^. Gradually, from the prodromal stage of MCI, AD progresses to dementia. Studies indicate that AD develops in patients with MCI at a rate of 10–15% per year^3^. Early identification of MCI patients can halt or delay the progression from the MCI stage to AD. Patients in the intermediate phases of MCI exhibit subtle morphological variations in their brain lesions^3^.

Recent studies highlight that early mild cognitive impairment (EMCI) manifests in the initial stages of MCI. In contrast, late mild cognitive impairment (LMCI) or progressive mild cognitive impairment (PMCI) denotes symptoms that deteriorate over time^4^. As symptoms progress and transition between stages, medical professionals exercise greater caution^5^. Determining variations in specific symptoms across different sets can pose challenges for researchers. Various medical imaging modalities, such as positron emission tomography (PET)^6^, magnetic resonance imaging (MRI), and computed tomography (CT)^7^, offer standard testing formats and images essential for these modalities' experimental processes.

MRI stands out as an effective and safe instrument, widely recognized for diagnosing a range of diseases including brain tumors^8^, neurological disorders^9^, spinal cord injuries and abnormalities^10^, and liver diseases^11^. This versatility is attributed to its high sensitivity, facilitating early disease detection. Different MRI sequences possess unique capabilities suited for various disorders. In comparison to other modalities, MRI images are frequently utilized for AD classification^12^. Nonetheless, various features extracted from MRI images aid in the categorization and diagnosis of MCI or AD, including grey and white matter volumes, cortical thickness, and cerebral spinal fluid (CSF) levels, helping determine the disease stage^13^. Pre-trained CNNs have recently shown promise in automatically diagnosing cognitive illnesses from brain MR images. Notable deep neural networks previously trained and applied to MRI data encompass Alex-Net^14^, VGG16^15^, ResNet-18^16^, ResNet-34^17^, ResNet-50^18^, as well as Squeeze-Net and InceptionV3^19^.

Typically, enhancing existing deep networks^16,20^ may not always address the low transfer efficiency stemming from disparities between medical and non-medical images. Furthermore, numerous factors can contribute to overfitting and inefficient utilization of space. To distinguish between patients with AD, EMCI, MCI, LMCI, and those cognitively normal (CN), we propose an innovative approach for developing CNN models, achieving high accuracy in multi-class classification tasks, especially for MRI categorization.

The major contributions of our paper include:

1. **New CNN Model Architecture:** We introduce two simplified CNN models, each possessing a straightforward structure. Despite their simplicity, these models achieve approximately 95% accuracy in the 5-way classification problem, illustrating that effective models can be designed without excessive complexity.
2. **Filter Size Impact:** Our study demonstrates that reducing filter size can yield improved classification outcomes. For instance, CNN2, using a 5 × 5 filter size, requires twice the number of filters of CNN1, with a 3 × 3 filter size, to attain similar accuracy levels.
3. **Concatenation Technique:** We introduce a novel approach by combining our two evolving CNN models at the classification layer, diverging from prior methods that integrate pre-trained models^18,19,21,22^. Our concatenation approach boosts accuracy from 95 to 99.13% in the 5-way classification task, offering dual benefits: enabling models to learn task-specific features and complementing each other's capabilities.
4. **Multi-Class Classification Performance:** We extend our methodology to address multi-class classification challenges, a departure from many studies focusing on binary or singular categories within multi-class problems. Utilizing MRI ADNI data, we apply our approach to 3-way, 4-way, and 5-way classification tasks, achieving outstanding accuracy rates of 99.43%, 99.57%, and 99.13%, respectively, underscoring the adaptability and reliability of our strategy across diverse classification scenarios.
5. **Comparative Analysis:** Leveraging MRI data, our research conducts an exhaustive comparative analysis between our proposed method and prevailing techniques for AD detection. This study elucidates the superiority or advancements of our approach over prior methods, benchmarked against accuracy metrics.

The subsequent sections are organized as follows: section “Related work” presents the most recent studies on early AD detection. Section “Materials” delineates the dataset employed in our research and its preparation methodology. Section “The proposed CNN model description” outlines our recommended model for AD diagnosis. Section “Discussion” unveils experimental outcomes on the ADNI dataset, accompanied by comprehensive discussions and juxtapositions with prior research. Finally, section “Conclusion” encapsulates our conclusions.

## Related work

In recent years, there has been a surge in the application of deep learning techniques to categorize Alzheimer’s disease (AD) using data from multimodal brain imaging. Leveraging the rich data provided by numerous imaging modalities, several research studies have proposed enhanced deep convolutional neural networks (CNNs) for AD categorization.

For predicting MCI conversion, the authors of^23^ developed a domain transfer learning-based model. They utilized various modalities, employing target and auxiliary domain data samples. Following experimental procedures, they employed domain transfer learning, achieving a prediction accuracy of 79.40%. Reference^24^ introduced a robust deep-learning methodology using MRI and PET modalities. They incorporated a dropout strategy to enhance performance in terms of categorization. Additionally, they applied the deep learning framework's multi-task learning method, assessing variations with and without dropout. The dropout technique yielded experimental findings indicating a 5.9% improvement. In^25^, the authors presented two CNN-based models, evaluating volumetric and multi-view CNNs in classification tests and integrating multi-resolution filtering, which directly influenced classification outcomes.

The authors of^26^ proposed a 2D CNN method based on ResNet50, incorporating multiple batch normalization and activation algorithms to classify brain slices into three classes: NC, MCI, and AD. The proposed model achieved an accuracy rate of 99.82%. To identify specific local brain morphological traits essential for AD diagnosis, another study^27^ developed a SegNet-based deep learning approach, finding that employing a deep learning technique and a pre-trained model significantly enhanced classifier performance. In^28^, a 3D CNN was designed to distinguish between AD and CN using resting-state fMRI images. Meanwhile, Çelebi et al.^29^ utilized morphometric images from Tensor-Based Morphometry (TBM) preprocessing of MRI data. Their study employed the deep, dense block-based Xception architecture-based DL method, achieving high accuracy in early-stage Alzheimer's disease diagnosis. However, this study did not address issues such as dataset variability, overfitting, and challenges with TBM image feature extraction.

To diagnose Alzheimer’s disease, Baglat et al.^30^ proposed hybrid machine learning-based models using SVM, Random Forest, and logistic regression. Their models utilized MRI patient scans from the OASIS dataset. Salehi et al.’s^31^ analysis emphasized that employing a deep learning approach would enhance early-stage Alzheimer’s disease forecasting. They utilized the OASIS and ADNI datasets, respectively. Fu’adah et al.^20^ introduced an AlexNet-based CNN classification model, achieving 95% accuracy using a collection of MRI images related to Alzheimer’s.

Murugan et al.^32^ presented a CNN model for Alzheimer’s disease recognition. Their proposed model consisted of two convolutional layers, one max-pooling layer, and four dementia network blocks, achieving an accuracy of 95.23% using the ADNI MRI image dataset. Salehi et al., in another study, employed MRI scans to diagnose Alzheimer’s disease using a CNN, achieving an average accuracy of 84.83%. Concurrently, Noh et al.^33^ proposed a 3D-CNN-LSTM model, utilizing extractors for spatial and temporal features and achieving high accuracy results of 96.43%, 95.71%, and 91.43%.

Rallabandi et al.^34^ presented a system for early diagnosis and categorization of AD and MCI in older cognitively normal individuals, employing the ADNI database. Their model achieved a 75% accuracy across various machine learning techniques. Furthermore, Odusami et al.^21^ introduced a pre-trained CNN hybrid model, employing deep feature concatenation, weight randomization, and gradient-weighted class activation mapping to enhance Alzheimer’s disease identification. Bamber et al.^35^ developed a CNN using a shallow convolution layer for Alzheimer’s disease classification in medical image patches, achieving an accuracy of 98%. Additionally, Akter et al.’s AlzheimerNet, a modified InceptionV3 model^36^, demonstrated outstanding accuracy in Alzheimer's disease stage classification from brain MRIs, surpassing traditional methods with a test accuracy of 98.67%.

## Materials

This section demonstrates the data source used to train a CNN model to recognize AD phases and the preprocessing image methods applied to the dataset.

### Description of the AD dataset

On the internet, numerous datasets can be used to classify AD. However, some of the CSV-formatted AD datasets are inappropriate for this study. Access to datasets from dedicated organizations such as Kaggle, ADNI^37^, and OASIS^38^ is available for research and educational purposes. The MRI ADNI dataset contains the MRI scans utilized in this study. The Alzheimer's Disease Neuroimaging Initiative (ADNI) dataset includes patients with Alzheimer's disease, mild cognitive impairment (MCI), and healthy controls. The ADNI dataset encompasses genetic information, cognitive tests, blood and CSF biomarkers, MRI and PET images, as well as clinical information. Table 1 presents statistical information regarding the MRI ADNI dataset.

**Table 1:** Key statistics for each clinical diagnosis.

Class	Number of Subjects	Average age	Average education level	Average hippocampus volume	Average fractional anisotropy (FA) of the corpus callosum	Average mean diffusivity (MD) of the White Matter	Gender distribution
AD	171	76.2 years	15.8 years	4.5 cubic centimeters	0.65	0.85	52.8% Female, 47.2% male
LMCI	72	72.3 years	16.4 years	5.1 cubic centimeters	0.68	0.80	
MCI	233	71.5 years	16.6 years	5.5 cubic centimeters	0.70	0.78	
EMCI	240	69.3 years	16.9 years	5.8 cubic centimeters	0.72	0.76	
CN	580	70.1 years	16.8 years	6.0 cubic centimeters	0.73	0.75	

**Figure 1:**
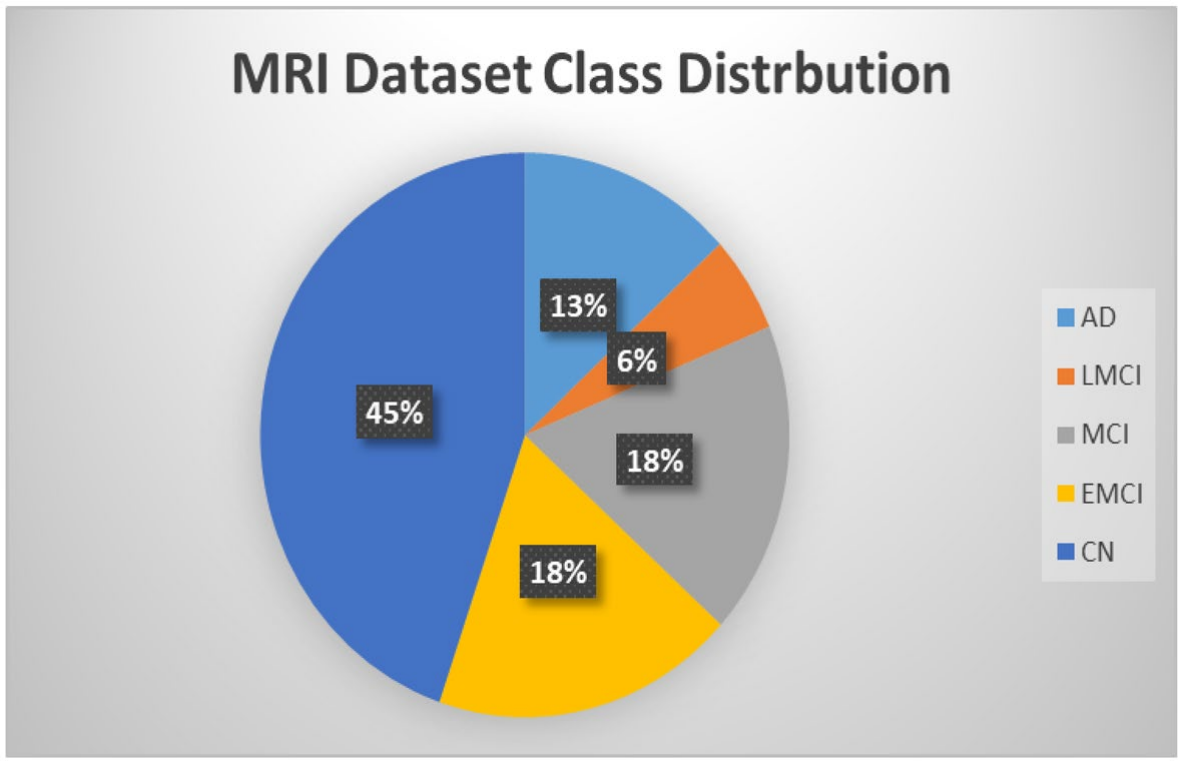
Class distribution of the MRI dataset.

This data consists of 1296 T1-weighted MRI scans. Each scan produces a 3D picture of the brain with a resolution of 1.5 mm isotropic voxels. As seen in Fig. 1, the scans are classified into one of five classes: CN patients, EMCI, LMCI, AD, and MCI.

### Data preprocessing

**Figure 2:**
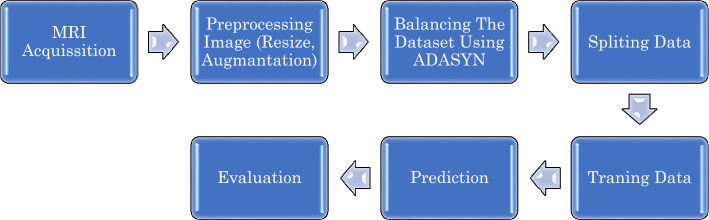
The methodology of the proposed work.

The ADNI dataset was chosen for this study based on its suitability for our research objectives. The ADNI dataset, contributed by the Alzheimer’s Disease Neuroimaging Initiative (ADNI), represents a globally collaborative research effort aimed at developing and validating neuroimaging tools to track the progression of Alzheimer's disease (AD). This dataset comprises data collected from ADNI Imaging Centers, located in clinics and medical institutions across the United States and other parts of the world. Prior to its public release, the data underwent processing and preparation by ADNI-funded MRI Analysis Laboratories. To optimize the quality and consistency of the images for analysis, the dataset's images underwent essential pre-processing steps. As illustrated in Fig. 2, these steps included:

- **Scaling:** Uniformly resizing all images to 224 pixels in both width and height.
- **Augmentation:** Enhancing the dataset’s diversity and mitigating overfitting by employing data augmentation techniques, as referenced in^39,40^.

To address the issue of imbalanced classes within the dataset, as visualized in Fig. 1, we employed the ADASYN technique to generate synthetic data for underrepresented classes.

#### Data augmentation

To minimize overfitting during neural network training, data augmentation is employed. This technique involves making class-preserving changes to individual data, artificially expanding the dataset^41^. Using methods that ensure replicability allows for the generation of new samples without altering the image’s semantic meaning. Given the challenges of manually locating newly labeled photos in the medical field and the limited availability of expert knowledge, data augmentation emerges as a reliable method to expand the dataset.

For our work, we devised an image augmentation method that incorporates cropping, scaling, flipping, and adjusting the brightness and contrast of the images.

#### ADASYN technique for balancing the AD dataset

There are two standard resampling methods: oversampling and under sampling. Oversampling creates samples for the minority class, while under sampling reduces samples from the majority class. In the proposed strategy, we employ an oversampling technique called ADASYN^42^. ADASYN stands for Adaptive Synthetic Sampling Approach, a technique in machine learning designed to address class imbalance in datasets. Like SMOTE (Synthetic Minority Oversampling Technique), ADASYN aims to enhance the performance of classification models by artificially increasing the number of data points in the minority class. However, ADASYN employs a more sophisticated approach than SMOTE.

**Figure 3:**
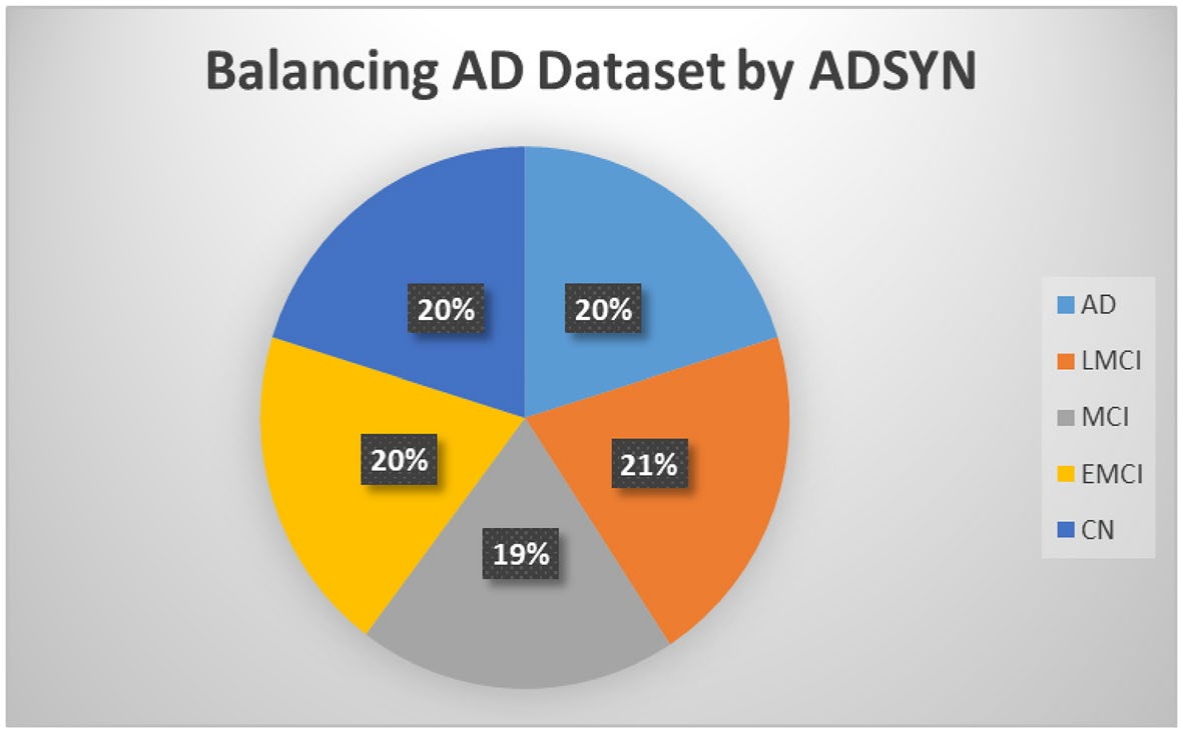
Class distribution of the MRI dataset after oversampling.

The core concept of ADASYN involves using weighted distributions for different minority-class examples based on the difficulty the learner faces in understanding them. This creates more comprehensive data for the more challenging minority-class instances compared to the easier-to-understand minority-class examples. Thus, the ADASYN approach enhances understanding of data dispersion in two ways: it mitigates bias stemming from class imbalance and adaptively focuses classification inference on complex samples. As depicted in Fig. 3, to better represent the minority classes, ADASYN introduces additional synthetic examples using nearest-neighbor methods, whereas SMOTE merely duplicates existing minority class points, potentially leading to overfitting. Conversely, ADASYN strategically generates new data points in areas where they're most needed, potentially yielding improved performance. Therefore, ADASYN outperforms SMOTE in handling complex data and reducing overfitting.

### Data splitting

**Figure 4:**
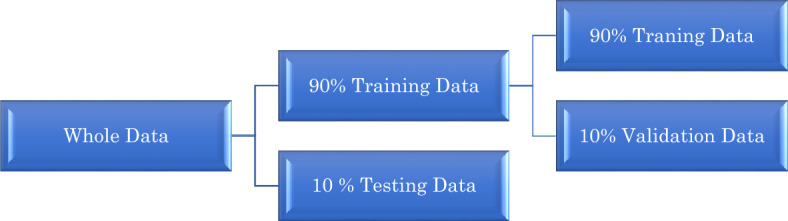
Schematics representation of the data splitting.

In this approach, the dataset was divided into three subsets. The training and validation sets are used to evaluate model performance by training on data, while the test data subset is employed for model prediction. As depicted in Fig. 4, the data was randomly allocated, with 90% for training and 10% for testing. Subsequently, cross-validation was applied solely to the training data. This process involves dividing the data into multiple subsets, evaluating each subset as a validation set, and then averaging the outcomes. Such an approach helps alleviate potential dataset bias. The validation dataset assists in selecting hyper-tuning parameters, such as regularization and learning rate. Proper hyper-tuning can mitigate overfitting and enhance accuracy. Once the model runs effectively with the validation subset, it stops training after a specific period to prevent redundant experiments.

Upon completing the learning process, the model underwent testing using a distinct test set. This particular test set remained untouched during the training phase, ensuring no overlap between the training and test data. It was exclusively reserved to assess the model's performance, calculating various metrics like accuracy, precision, recall, or other evaluation measures that gauge the model's ability to generalize to unseen data.

## The proposed CNN model description

**Figure 5:**
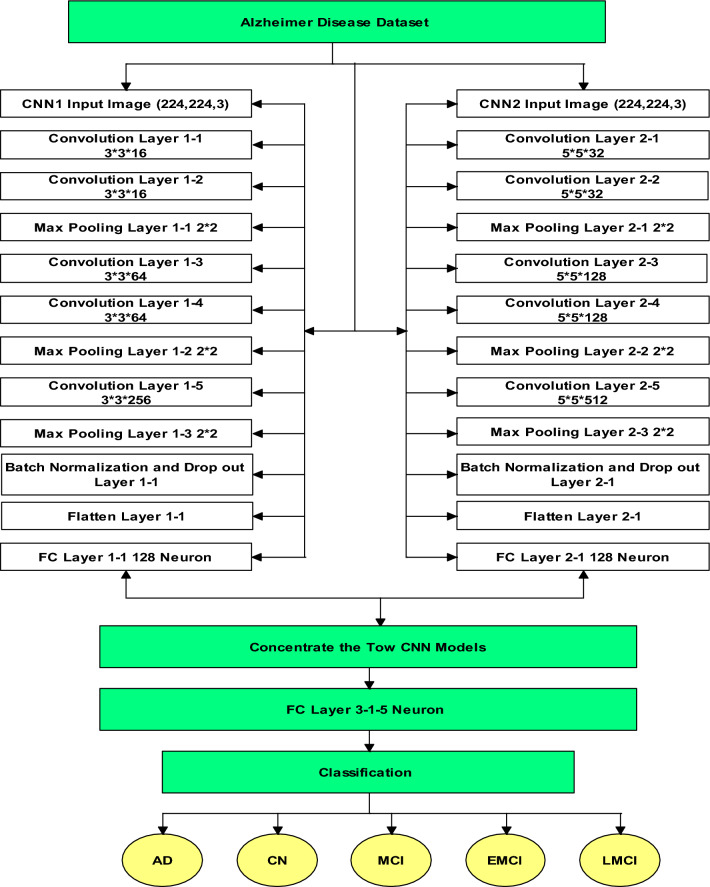
The proposed CNN architecture.

To process diverse patient data, we are constructing a network comprising two separate CNN models concatenated in a classification layer, as illustrated in Fig. 5. A 224 × 224 × 3 tensor, representing the temporal dimension and the axes (x, y, and z), serves as the input for the network. The first CNN model is initiated with two convolutional layers, each housing 16 filters of size 3 × 3.

These filters extract local features from the input images. Subsequently, 2 × 2 max-pooling layers with a stride of 2 are applied to down sample the feature maps and capture pivotal information. The subsequent two convolutional layers each incorporate 64 filters, enhancing the representation of higher-level features. Another round of max-pooling is executed to reduce spatial dimensions. Following this, a single convolutional layer with 256 filters of size 3 × 3 is introduced to capture intricate patterns. To combat overfitting, a dropout layer with a 20% rate is incorporated, and batch normalization is employed to normalize activations, ensuring improved training stability. Finally, a fully connected layer with 128 neurons is appended to glean global insights from the flattened feature maps.

The second CNN model follows a comparable structure but with distinct filter sizes. It commences with two convolutional layers, each comprising 32 filters of size 5 × 5. Subsequently, 2 × 2 max-pooling layers are applied with a stride of 2. The ensuing two convolutional layers each contain 128 filters of size 5 × 5. A subsequent round of max-pooling is executed for spatial dimension reduction. This is succeeded by a convolutional layer encompassing 512 filters of size 5 × 5. Similarly, a 20% dropout layer is employed to prevent overfitting, and batch normalization is integrated for enhanced training stability. Ultimately, a fully connected layer with 128 neurons is appended to extract global insights from the feature maps.

Prediction, denoting the probability that the input belongs to any of the five classes, is generated by concatenating features extracted from each CNN network and processing the outcomes on a Fully Connected network. The predicted class is then determined based on the highest value. Table 2 furnishes a comprehensive description of the network architecture, detailing each convolutional layer’s operations, size, filter count, and output. Additionally, the parameters for each layer are enumerated. Each parameter is trainable, integrated into the backpropagation process, while Table 3 enumerates the CNN model’s hyperparameters created.

**Table 2:** The proposed CNN parameter.

Layer (type)	Output shape	Parameter
Input layer 1	[(None, 224, 224, 3)]	0
Input layer 2	[(None, 224, 224, 3)]	0
Convolution layer 1–1	(None, 222, 222, 16)	448
Convolution layer 2–1	(None, 220, 220, 32)	2432
Convolution layer 1–2	(None, 220, 220, 16)	2320
Convolution layer 2–2	(None, 216, 216, 32)	25,632
Max pooling layer 1–1	(None, 110, 110, 16)	0
Max pooling layer 2–1	(None, 108, 108, 32)	0
Convolution layer 1–3	(None, 108, 108, 64)	9280
Convolution layer 2–3	(None, 104, 104, 128)	102,528
Convolution layer 1–4	(None, 106, 106, 64)	36,928
Convolution layer 2–4	(None, 100, 100, 128)	409,728
Max pooling layer 1–2	(None, 53, 53, 64)	0
Max pooling layer 2–2	(None, 50, 50, 128)	0
Convolution layer 1–5	(None, 51, 51, 256)	147,712
Convolution layer 2–5	(None, 46, 46, 512)	1,638,912
Max pooling layer 1–3	(None, 25, 25, 256)	0
Max pooling layer 2–3	(None, 23, 23, 512)	0
Batch normalization and drop out layer 1–1	(None, 25, 25, 256)	0
Batch normalization and Drop out layer 2–1	(None, 23, 23, 512)	0
Flatten layer 1–1	(None, 160000)	0
Flatten layer 2–1	(None, 270848)	0
FC layer 1–1	(None, 128)	20,480,128
FC layer 2–1	(None, 128)	34,668,672
Concatenate	(None, 256)	0
FC layer 3–1	(None, 5)	1285
Total parameters: 57,526,005		
Trainable parameters: 57,526,005

**Table 3:** The developed CNN model hyper-parameters.

Activation function	ReLU
Dropout rate	.2
Optimizer	Adam
No. of epoch	25 with early stop
Classifier	SoftMax
Loss function	Categorical Cross-entropy

Numerous variants were evaluated to ascertain the suitability of different layers and certain hyperparameters utilized in the network. These evaluations encompassed batch normalization, various dropout rates, and diverse pooling techniques.

### Performance evaluation metrics

The test set, created by partitioning the original dataset before training the model, was utilized to evaluate the model. The robustness of the model has been ensured using multiple metrics^43^. The efficacy of the model's training is gauged by how comprehensively these metrics are interpreted. We employed a variety of indicators to assess the performance of our model.

1. **Accuracy:** Accuracy represents the percentage of actual forecasts that were correctly predicted. Generally, values above 80% are considered good, while values exceeding 90% are deemed excellent. This metric is determined by the following expressions^43^.
  1 $${\varvec{accuracy}} = \frac{{{\varvec{TP}} + {\varvec{TN}}}}{{{\varvec{TP}} + {\varvec{TN}} + {\varvec{FP}} + {\varvec{FN}}}}$$
  where, ***TP, TN*, *FN*, *FP*** are **True Positive**, **True Negative**, **False Negative**, and **False Positive values**, respectively.
2. **Precision:** The following equation is used to compute precision, which is defined as the ratio of accurate optimistic forecasts to all optimistic predictions^46^. In general, precision values over 80% are regarded as satisfactory.
  2 $${\varvec{precision}} = \frac{{{\varvec{TP}}}}{{{\varvec{TP}} + {\varvec{FP}}}}\user2{ }$$
3. **Recall**: It can also be referred to as the sensitivity score or true positive rate. Recall involves contrasting accurate optimistic predictions with all actual correct positives^43^. Acceptable recall values typically range from 70 to 90%. The following equation is used to compute the recall:
  3 $${\varvec{Recall}} = \frac{{{\varvec{TP}}}}{{{\varvec{TP}} + {\varvec{FN}}}}$$
4. **F1-score:** The F1 score is remarkable in that it provides a distinct value for each class label^43^. Use the following calculation to determine the F1-score.
  4 $${\varvec{F}}1 - {\varvec{Score}} = 2 \times \frac{{{\varvec{Precision}} \times {\varvec{Recall}}}}{{{\varvec{Precision}} + {\varvec{Recall}}}}$$
5. **Balanced accuracy:** It is calculated by averaging the true positive rate (TPR) and true negative rate (TNR). The TPR represents the ratio of positive to adverse events accurately identified, while the TNR signifies the ratio of negative to positive events^44^.
6. **Matthews Correlation Coefficient (MCC):** The MCC is a more complex metric that considers the imbalance between positive and negative examples in a dataset. If one class significantly outweighs the other in occurrences, the metric can become uneven^45^. The MCC is calculated as follows:
  5 $${\varvec{MCC}} = \frac{{\left( {{\varvec{TP}} \times {\varvec{TN}}} \right) - \left( {{\varvec{FP}} \times {\varvec{FN}}} \right)}}{{\sqrt {\left( {{\varvec{TP}} + {\varvec{FP}}} \right)\left( {{\varvec{TP}} + {\varvec{FN}}} \right)\left( {{\varvec{TN}} + {\varvec{FP}}} \right)\left( {{\varvec{TN}} + {\varvec{FN}}} \right)} }}$$

### Model development and training

In our work, we trained and validated the classifier using open-source software: Python 3.0 and the Google Collaboratory Pro platform^46^, equipped with a GPU: 1xTesla K80, featuring 2496 CUDA cores and a compute capability of 3.7. It has 12 GB of GDDR5 VRAM (11.439 GB usable). To develop our proposed model, we chose to utilize the Keras library integrated with TensorFlow modules. Additionally, we employed Python libraries such as Scikit-learn, Numpy, and OpenCVas Python libraries.

## Experiments and results

In the following section, we delve deeply into the steps of the experiment, present the results, and compare them with previous findings.

As depicted in Fig. 2, after loading the ADNI MRI data, we augmented the images and utilized the ADASYN approach to address data imbalance. The dataset size expanded to 3,000 images post ADASYN application. Subsequently, we divided the data into three sets based on the proportions illustrated in Fig. 3: training, validation, and test sets. Ultimately, we used the training data to train the proposed model.

The proposed model comprises two distinct CNNs merged at the classification stage. We applied the 5-way multiclass MRI dataset to each network individually. Performance evaluation employed metrics such as accuracy, recall, precision, balanced accuracy, Matthew's correlation coefficient, and loss function. These individual network performances were then juxtaposed with the combined CNN performance, as outlined in Table 4.

**Table 4:** The performance of the first developed CNN, the second developed CNN and the proposed model for test data.

Metrics	CNN1	CNN2	Proposed
Loss	0.3286	0.1491	0.0325
Accuracy	95.42%	95.77%	99.30%
Recall	95.07%	95.77%	99.30%
Precision	95.74%	96.11%	99.30%
Balanced accuracy	95.45%	95.82%	99.32%
Matthew’s correlation coefficient	94.29%	94.67%	99.13%

Tables 5, 6, and 7 present the classification performance results of these CNN networks, focusing on metrics like recall, precision, f1-scores, and support, where 'support' denotes the number of samples.

**Table 5:** The result of Precision, Recall, and F1-Score for each class when Appling the first developed CNN only on the test data to classify it in to 5 categories.

Classes	Precision	Recall	F1-Score	Support
AD	0.98	1.00	0.99	63
CN	0.90	0.91	0.90	57
EMCI	0.95	0.98	0.96	53
LMCI	1.00	1.00	1.00	53
MCI	0.94	0.88	0.91	58

**Table 6:** The result of Precision, Recall, and F1-Score for each class when applied the second developed CNN only on the test data to classify it in to 5 categories.

Classes	Precision	Recall	F1-Score	Support
AD	0.98	1.00	0.99	63
CN	0.93	0.95	0.94	57
EMCI	0.96	0.89	0.92	53
LMCI	1.00	1.00	1.00	52
MCI	0.92	0.95	0.93	59

**Table 7:** The result of Precision, Recall, and F1-Score for each class when applied the proposed CNN on the test data to classify it in to 5 categories.

Classes	Precision	Recall	F1-Score	Support
AD	0.98	1.00	0.99	58
CN	0.93	0.95	0.94	59
EMCI	0.96	0.89	0.92	56
LMCI	1	1	1	55
MCI	0.92	0.95	0.93	58

As you can see, reducing the size of a filter can lead to improved classification results. Specifically, CNN2, which employs a 5 × 5 filter size, needs to utilize twice the number of filters present in CNN1 (which uses a 3 × 3 filter size) to achieve a comparable accuracy to CNN1. Furthermore, when the two networks are combined, the resultant network exhibits higher accuracy than either of the individual networks. This improvement arises because the two networks complement one another, offering different perspectives on the data.

To evaluate the effectiveness of this approach across various classification tasks, we applied the combined network to datasets, providing experimental results for a benchmark five-way multiclass classification problem^16^, a benchmark four-way multiclass classification problem^28^, and a benchmark three-way classification problem^47^.

**Figure 6:**
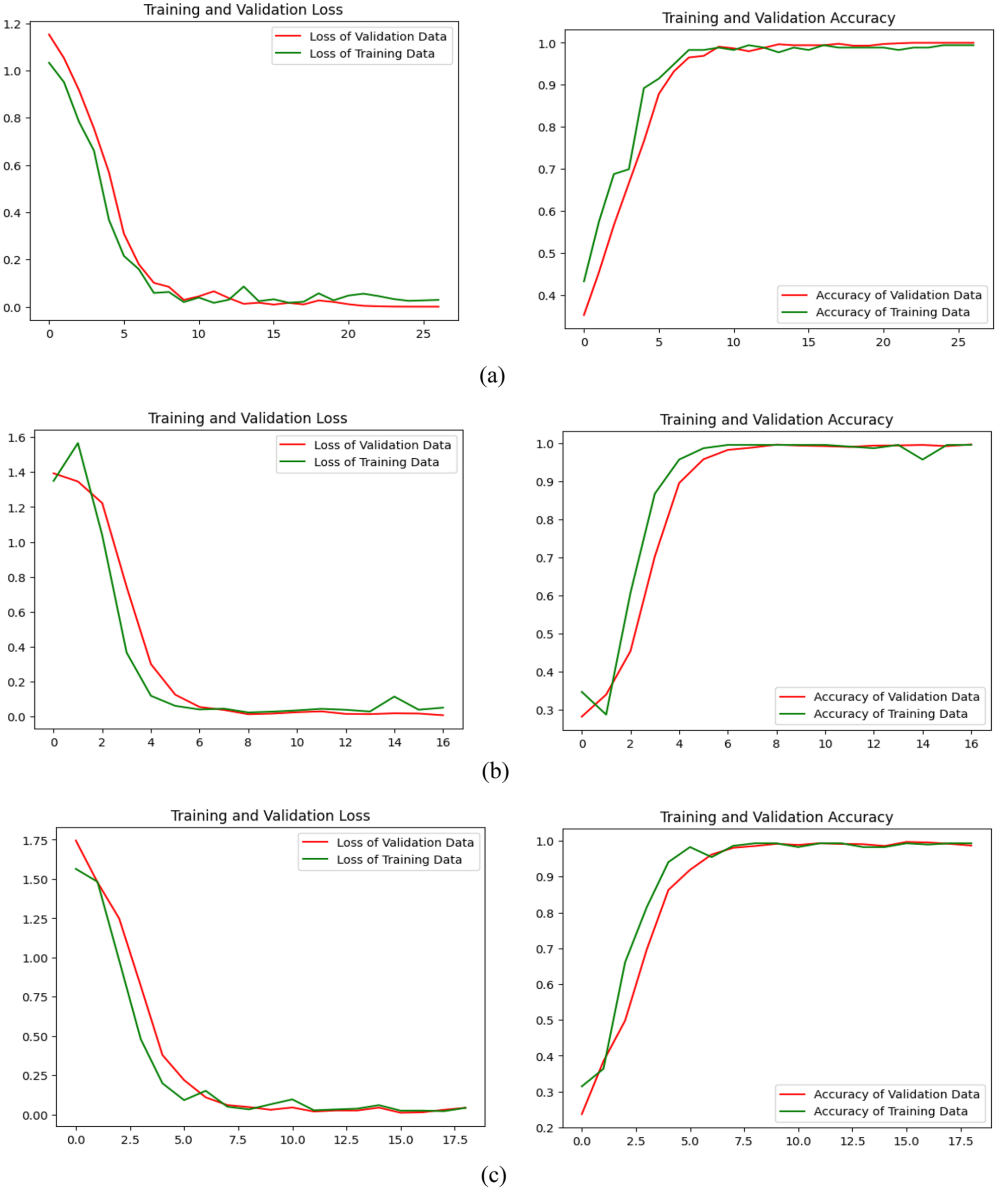
The training loss/validation loss and training accuracy/validation accuracy of the proposed model (**a**) 3-way multiclass; (**b**) 4-way multiclass; (**c**) 5-way multiclass.

In Fig. 6, we initially display graphs contrasting the proposed model’s training accuracy against validation accuracy, as well as training loss versus validation loss, for the three-way, four-way, and five-way multiclass problems. Table 8 juxtaposes the performance of the proposed model across the aforementioned multiclass problems.

**Table 8:** The performance of proposed model with 3-way multiclass; 4-way multiclass; and 3-way multiclass.

Metrics	3-way multiclass	4-way multiclass	5-way multiclass
Loss	0.0163	0.0414	0.0325
Accuracy	99.43%	99.57%	99.30%
Recall	99.43%	99.57%	99.30%
Precision	99.43%	99.57%	99.30%
Balanced accuracy	99.35%	99.57%	99.32%
Matthew’s correlation coefficient	99.15%	99.43%	99.13%

### Confusion matrix

It is employed to evaluate and compute various classification model metrics. It gives the numerical breakdown of a model’s predictions during the testing phase^43^.

**Figure 7:**
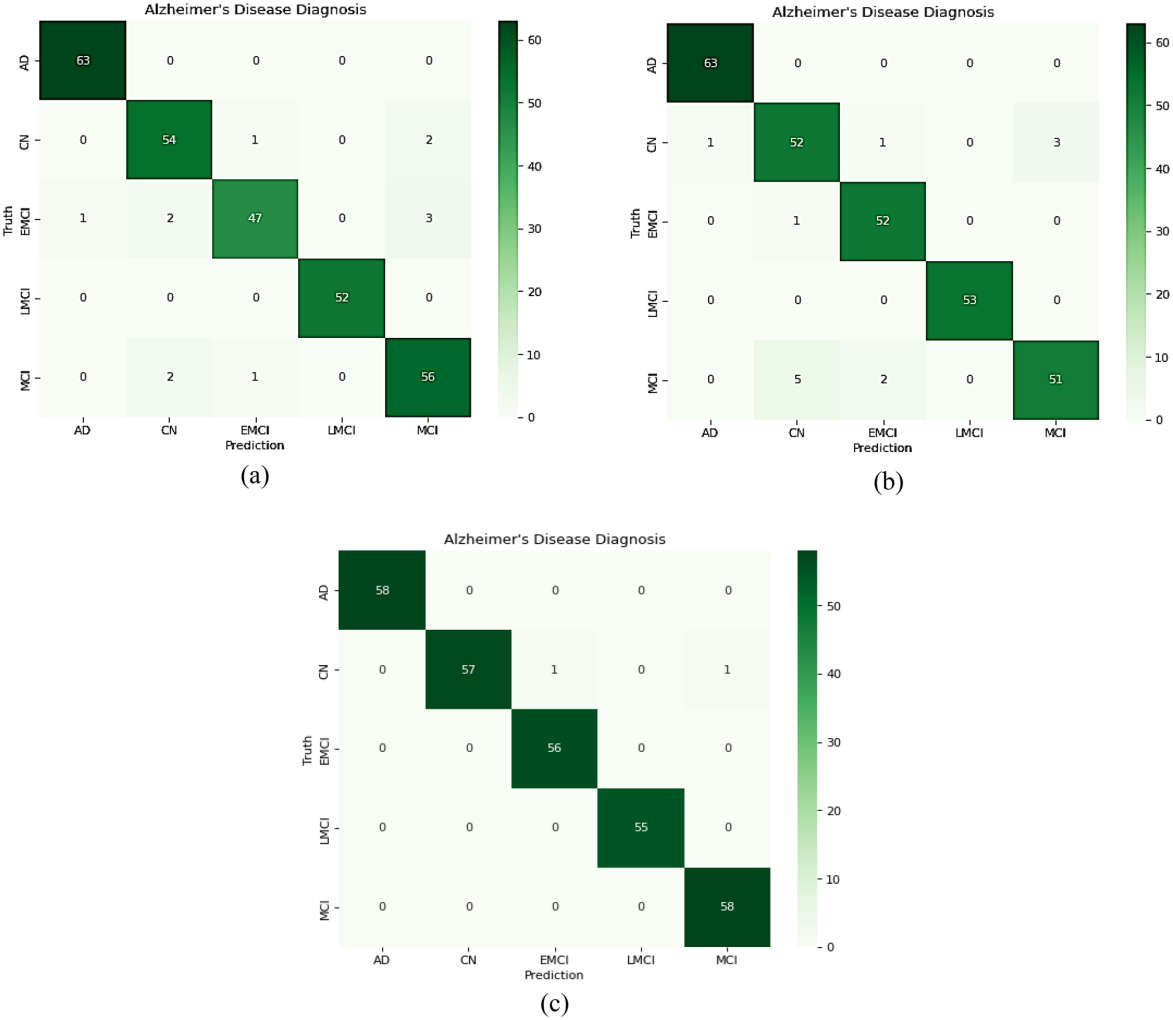
Confusion matrix of proposed model on test data (**a**) CNN1; (**b**) CNN2; (**c**) the overall developed CNN.

**Figure 8:**
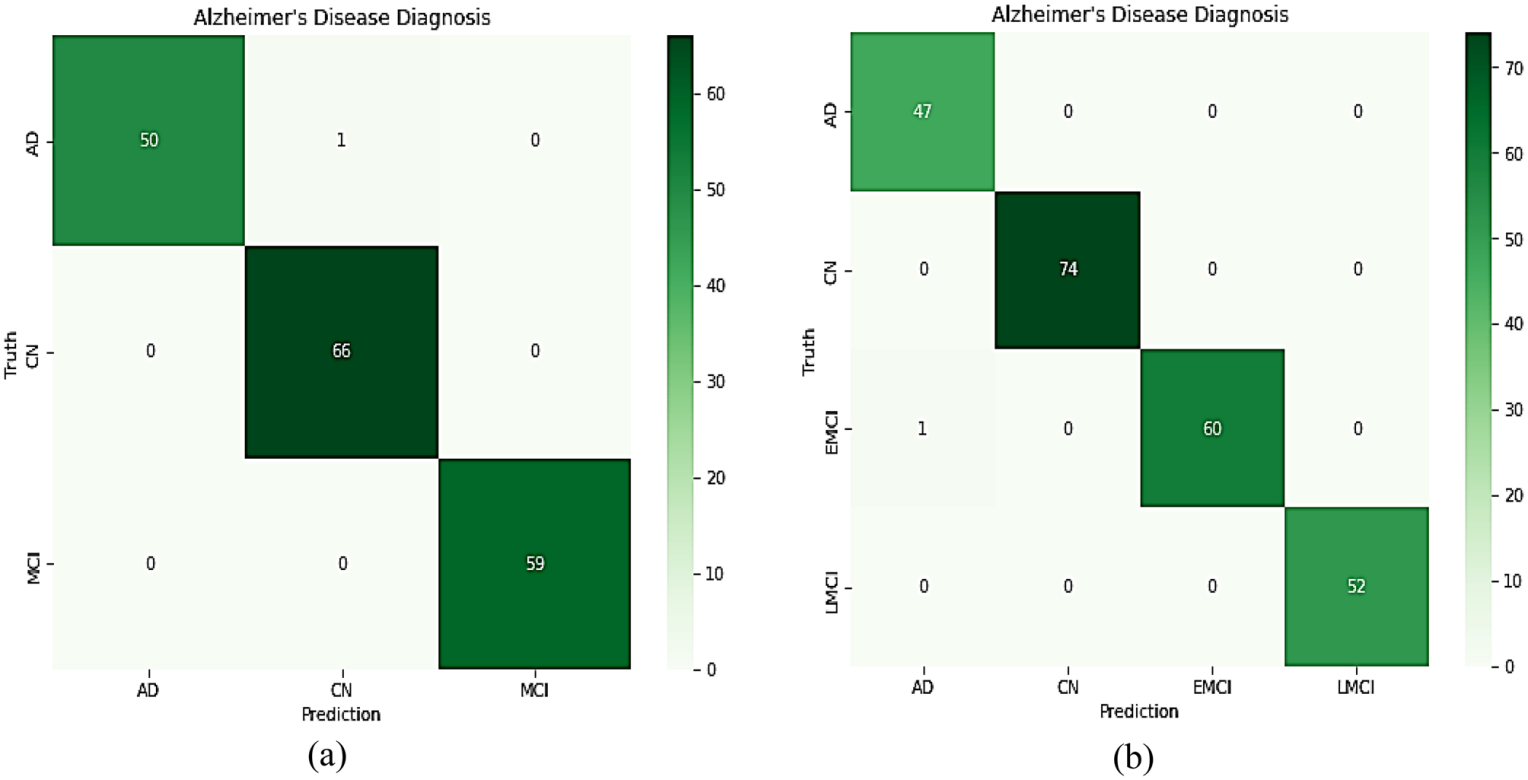
Confusion matrix of proposed model on test data (**a**) 5-way multiclass; (**b**) 4-way multiclass.

A Confusion matrix for the proposed model was developed, as seen in Figs. 7 and 8, to evaluate how well the suggested network performed on each class in the test data. Additionally, Tables 7, 9, and 10 provide specifics regarding the class classification report of the proposed model based on precision, recall, and F1-score.

**Table 9:** The result of Precision, Recall, and F1-Score for each class when applied the proposed CNN on the test data to classify it in to 3 categories.

Classes	Precision	Recall	F1-Score	Support
AD	1.00	0.98	0.99	51
CN	0.99	1.00	0.99	66
MCI	1.00	1.00	1.00	59

**Table 10:** The result of Precision, Recall, and F1-Score for each class when applied the proposed CNN on the test data to classify it in to 4 categories.

Classes	Precision	Recall	F1-Score	Support
AD	0.98	1.00	0.99	47
CN	1.00	1.00	1.00	74
EMCI	1.00	0.98	0.99	61
LMCI	1.00	1.00	1.00	52

Figure 7c shows that one subject of CN was misclassified as EMCI, and another was misclassified as MCI in the case of five multiclass classifications. This indicated an influential model because, in medical diagnosis, screening a person as diseased is preferred over eliminating a diseased person by falsely predicting a negative. As dedicated in Fig. 8, one subject of EMCI was incorrectly diagnosed with AD in four multiclass classifications. One EMCI was misclassified *as AD in a three-way multiclass.*

**Figure 9:**
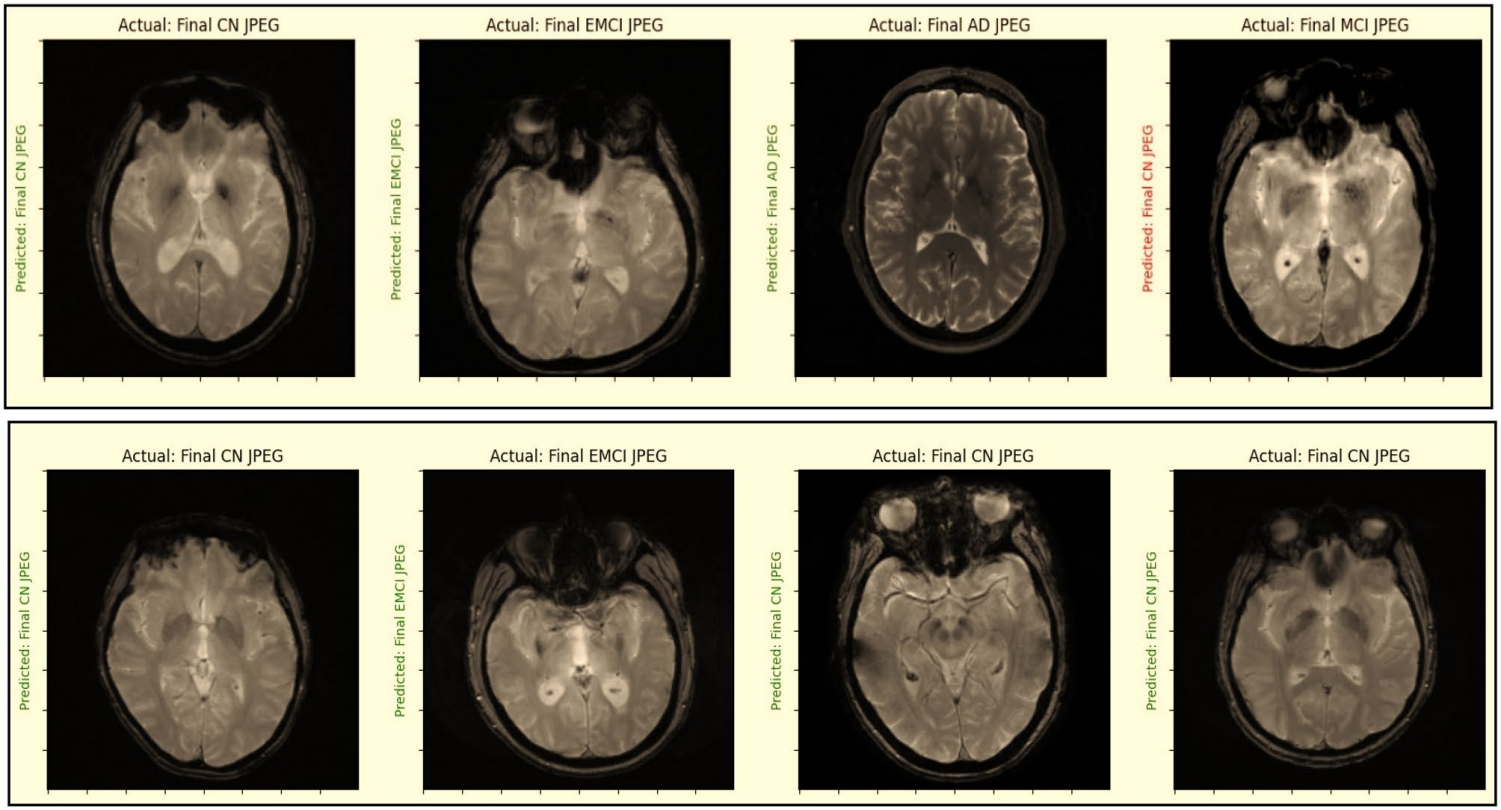
Examining the predicted label matched the real label or not.

For the three-way, four-way, and five-way multiclass classifications, the suggested model yielded average accuracy values of 99.43%, 99.57%, and 99.3%, respectively. Additionally, as depicted in Fig. 9, the suggested model was examined to determine whether the predicted label matched the actual label.

### GRAD-CAM analysis

**Figure 10:**
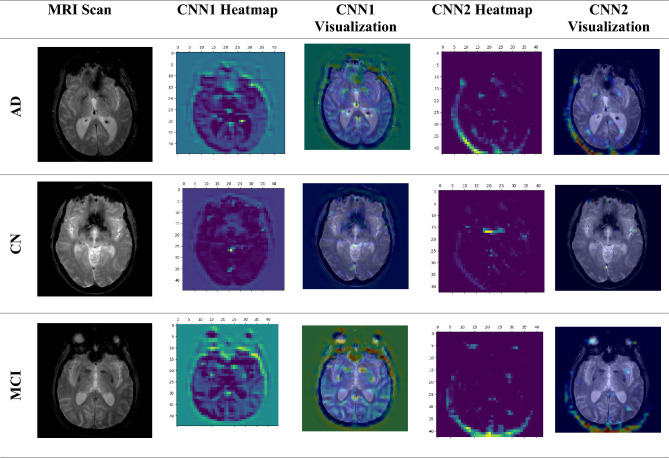
GRAD-CAM algorithm when apply to MRI scan images of an AD, CN, and MCI.

In the ongoing quest to understand and harness the power of deep learning, a crucial challenge lies in making these complex neural networks more interpretable. This is especially critical in applications like medical imaging, where trust and understanding are paramount. Deep learning can be shown in action with Gradient Weighted Class Activation Mapping (Grad-CAM), developed by Selvaraju et al.^48^. This ingenious technique acts as a magnifying glass for deep neural networks, providing a visual representation of their inner workings. It’s like peeking behind the curtain to see what these algorithms are focusing on when they analyze data. The MRI scan serves as the input for the suggested model, which is used as a detection technique. Grad-CAM is applied to the last convolution layer of the two proposed CNN models before concatenation has been used to get the expected label. The feature map for the suggested network is extracted in this case using the Grad-CAM technique. The heat map shows the image region that is essential for determining the target class as a visual depiction of a suggested network. Furthermore, the significance of every CNN model in decision-making as well as the impact of varying the size and quantity of filters in each model can be determined with this method. The heatmaps and visualizations created by applying the GRAD-CAM algorithm to MRI scan images of an AD, CN, and MCI are shown in Fig. 10. This visual evidence not only enhances our understanding of the model's predictions but also paves the way for validating Alzheimer’s diagnoses with greater confidence.

### ROC curve analysis

The proposed model’s performance is evaluated by computing the AUC (Area Under Curve) and ROC (Receiver Operating Characteristics Curve) values^49^. The single class vs. rest method is used for multiclass classification. ROC curves are built with 1-specificity (false positive rate) as the x-axis and sensitivity (true positive rate) as the y-axis. Calculating the area under the ROC curve yields the AUC score. The AUC value ranges from 0 to 1. The model's performance decreases as the value gets closer to 0. Likewise, the more closely the value approaches 1, the more well the model works.

**Figure 11:**
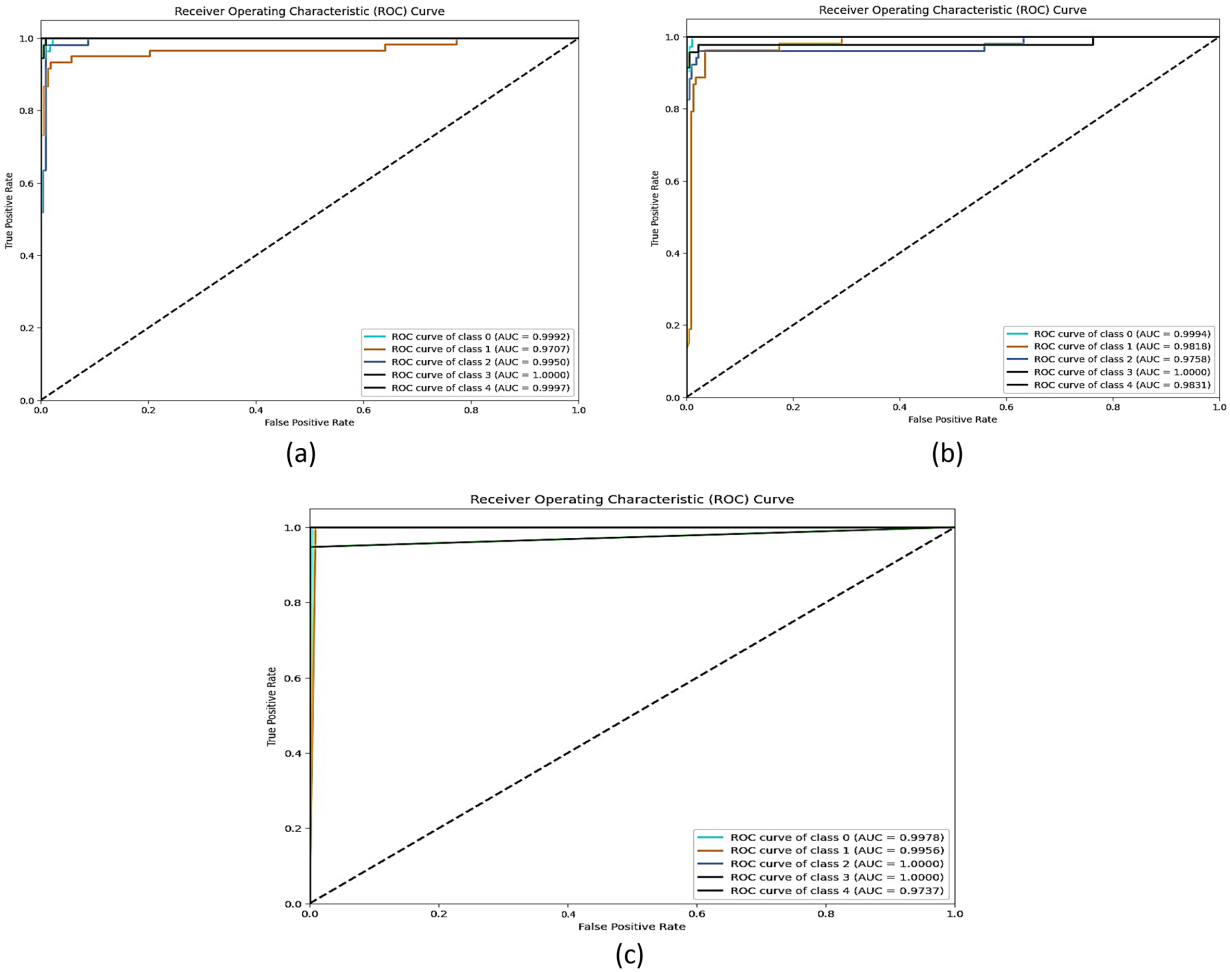
ROC curve and AUC value of the (**a**) CNN1, (**b**) CNN2, and (**c**) proposed model.

Figure 10 displays the ROC curves for the first, second, and suggested CNN models across the five classes. Taking into consideration that Classes 0, 1, 2, 3, 4, and 5 refer to CN, MCI, AD, LMCI, and EMCI, respectively. By examining Fig. 11, it can be observed the proposed model significantly improved the AUC values for all classes of Alzheimer's disease. The AUC value of the class CN is 0.9992, MCI is 0.9707, AD is 1, LMCI is 1, and EMCI is 0.9737. Whereas the AUC values when applying proposed CNN1 were as follows the class CN is 0.9978, MCI is 0.9956, AD is 0.9950, LMCI is 1, and EMCI is 0.9997. while the AUC values when applying proposed CNN2 were 0.9994 for CN, 0.9818 for MCI, 0.9758 for AD, 1 for LMCI, and 0.9831 for EMCI. Therefore, the proposed model is a more accurate and reliable method for diagnosing Alzheimer’s disease.

### Wilcoxon signed-rank test

To ensure that the results were not merely due to random chance, a significance statistical analysis (S) was conducted. The p-values for each model were computed, and the researchers utilized the Wilcoxon signed-rank test for this purpose. The Wilcoxon signed-rank test is commonly employed when comparing two non-parametric variables. Through this test, two independent samples are contrasted to assess pairwise differences across multiple observations from a single dataset. The outcome indicates whether there's a distinction in their population mean ranks. The p-values for the pairwise comparisons of the models^50,51^ are detailed in Table 11. Compared to the other models, the suggested model exhibited superior performance. In essence, the proposed model significantly outperformed the other four models, as indicated by the p-value difference between the suggested model and the others being less than 0.05.

**Table 11:** Wilcoxon Signed Rank test.

No.	Model pairwise comparisons	p-Value
1	Proposed model verses AlexNet	0.007280
2	Proposed model verses ResNet50	0.001655
3	Proposed model verses Xception	0.007157
4	Proposed model verses VGG16	0.002338

## Discussion

The findings revealed that the suggested model accurately distinguishes between the three-way multiclass (AD/MCI/CN), four-way multiclass (AD/CN/LMCI/EMCI), and five-way multiclass (AD/CN/LMCI/EMCI/MCI) categories of Alzheimer's disease.

Numerous studies have employed various methodologies to categorize the stages of AD. As shown in Table 12, we compared the performance of the proposed system with various models discussed in the literature review.

**Table 12:** Classification performance comparison.

Authors	Biomarker	Database	Methodology	Classification	Accuracy
Ramzan et al. (2020)^16^	MRI	ADNI	Resnet 18 (Finetuning)	5-way AD/CN/MCI/EMCI/LMCI	97.88%
Parmar et al. (2020)^28^	MRI	ADNI	3D CNN	4-way AD/CN/EMCI/LMCI	93.00%
Puete-Castro et al. (2020)^52^	MRI	ADNI	Resnet18 and SVM	3-way AD/CN/MCI	78.72%
Fu'adah et al. (2021)^20^	MRI	ADNI	AlexNet	4-way AD/CN/EMCI/LMCI	95%
Murugan et al. (2021)^32^	MRI	ADNI	CNN	4-way AD/CN/EMCI/LMCI	95.23%
Buvaneswari et al. (2021)^27^	Voxel-Based Morphometry (VBM)	ADNI	VGGNet GoogLeNet ResNet	Binary classification NC/AD	96.08% 97.15% 94.60%
Odusami et al. (2022)^21^	MRI	ADNI	Resnet18 and DenseNet121 with randomized weight	3-way AD/CN/MCI	98.21%
				4-way AD/CN/EMCI/LMCI	93.06%
				5-way AD/CN/MCI/EMCI/LMCI	98.86%
Noh et al. (2023)^33^	fMRI	ADNI	3D-CNN-LSTM classification model	4-way AD/CN/EMCI/LMCI	96.43%
Çelebi et al. (2023)^29^	Tensor-Based Morphometry (TBM)	ADNI	Xception architecture-based deep dense block	3-way AD/CN/MCI	95.81%
Akter et al. (2023)^36^	MRI	ADNI	Inception V3 (Finetuning)	6-way AD/CN/SMC /MCI/EMCI/LMCI	98.68%
Bamber et al., (2023)^35^	MRI	OASIS-3	CNN	4-way AD/CN/MCI/moderate MCI	98%
Proposed	MRI	ADNI	CNN	3-way AD/CN/MCI	99.43%
				4-way AD/CN/EMCI/LMCI	99.57%
				5-way AD/CN/MCI/EMCI/LMCI	

Clearly, the recommended approach yielded the best results in terms of accuracy and performed exceptionally well in 3-way, 4-way, and 5-way multiclass classification problems. Additionally, the results underscore the importance of concatenating multiple CNN models in the classification layer to enhance the model's discriminative ability. Compared to single-model techniques, our method excels in capturing AD-related patterns by integrating complementary data from different CNNs.

The proposed method offers several advantages over traditional methods for early AD detection:

1. While most classification approaches differentiate between images of AD and CN or AD and MCI, our study employs 3-way, 4-way, and 5-way multiclass categorizations.
2. Our emphasis lies in the early diagnosis of AD, achieved by enhancing the accuracy of distinguishing MCI, EMCI, LMCI, and CN.
3. Apart from the training data, we utilized independent sets of images to assess our model.

4. The suggested technique is non-invasive and is applicable to MRI scans, commonly used in clinical settings.
5. Our approach eliminates the need for manual feature extraction, a labor-intensive and subjective task.

Moreover, the proposed approach can extract intricate features from MRI images that are challenging to extract using conventional methods.

However, the suggested approach does have some limitations. First, the ADNI MRI dataset was utilized to evaluate our method. For the proposed strategy to be universally applicable, it should be tested on additional datasets. The extensive data required to train the CNNs in our approach could limit its clinical applicability in scenarios where data is scarce. Lastly, the model doesn’t incorporate clinical data; instead, it aids doctors in decision-making without replacing it.

In conclusion, our proposed strategy presents a promising avenue for the early detection of AD. This method could facilitate more timely and effective AD diagnoses, leading to improved therapeutic outcomes.

## Conclusion

**Figure 12:**
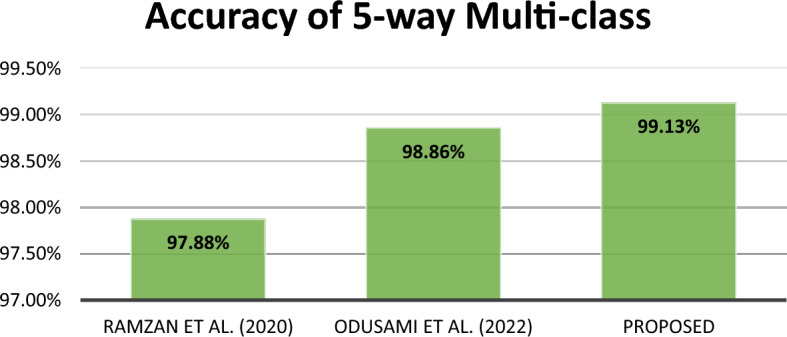
Accuracy comparison of different 5-way multi-class methods.

**Figure 13:**
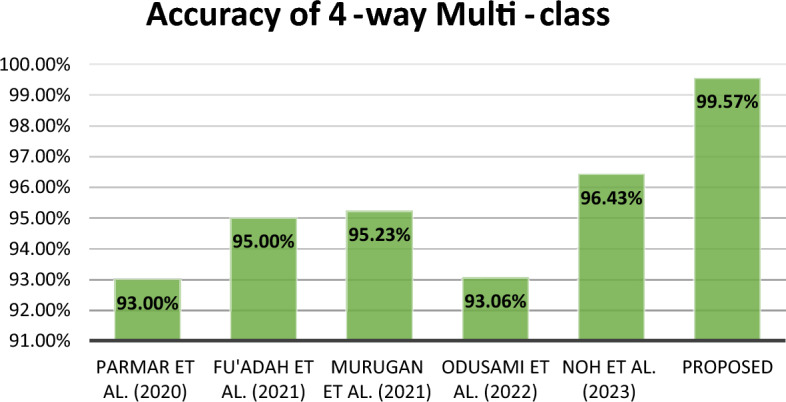
Accuracy comparison of different 4-way multi-class methods.

**Figure 14:**
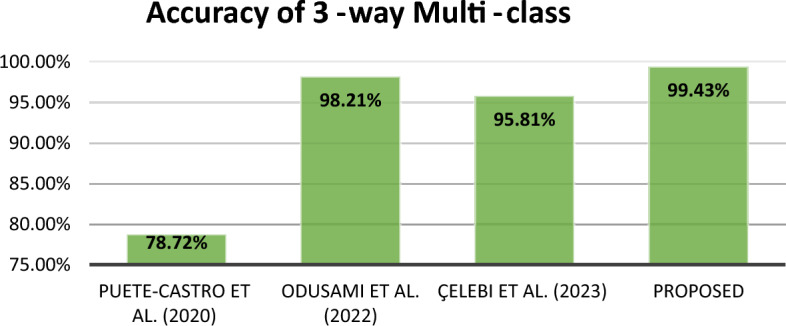
Accuracy comparison of different 3-way multi-class methods.

In summary, this research proposes a new method for early detection of Alzheimer's disease (AD) using magnetic resonance imaging (MRI) data. The suggested approach employs two convolutional neural networks (CNNs) and combines their outputs by concatenating them in a classification layer. The objective is to capture various spatial and structural features of the brain, facilitating a comprehensive analysis of AD-related patterns. The efficacy of our approach is demonstrated through experimental results on the ADNI dataset, as compared to findings from prior research, as depicted in Figs. 12, 13, and 14. For the 3-way, 4-way, and 5-way classification tasks, we achieved notably high accuracy rates of 99.43%, 99.57%, and 99.13%, respectively. Overall, this study advances the field of AD detection by introducing an innovative approach with promising accuracy results. The proposed method has the potential to assist doctors and researchers in earlier AD diagnosis, paving the way for proactive treatments and improved patient outcomes. Future endeavors will focus on validating the method with larger datasets, exploring its applicability in clinical settings, and integrating additional data modalities to enhance accuracy

### Supplementary Information

Supplementary Information.

## Data Availability

The MRI data used in my research is publicly available from the Alzheimer’s Disease Neuroimaging Initiative (ADNI) database^31^.

## Code availability

The Python code used for the processing is available upon request.
