# Supplementary material for: A novel CNN architecture for accurate early detection and classification of Alzheimer’s disease using MRI data
Source: Sci Rep. 2024 Feb 12;14:3463. doi: 10.1038/s41598-024-53733-6 (PMC10859371; doi:10.1038/s41598-024-53733-6)
Supplement: Supplementary file 1 — Supplementary Information. [file 41598_2024_53733_MOESM1_ESM.docx]

**Supplementary**

1. **Building Components of CNN**

Convolutional neural networks specialize in processing multidimensional data, including time series and images. They generate successive feature maps by aggregating basic data features, such as edges and vertices in images, from the initial layers into more intricate patterns like geometric shapes [53].

To produce these feature maps, convolutional operations with trainable kernels are applied to the input of the layer. Pooling and nonlinear transformations work together to facilitate the network's convergence. Subsequently, based on the processed feature maps, predictions are generated, often through fully connected layers.

In general, the standard architectural design for individual CNN models consists of five layers: the convolutional layer, max-pooling layer [54], batch normalization [55], dropout layer, and fully connected layer. The subsequent sections provide more detailed information about these components [56].

1. **The Convolutional Layer:** This layer discerns the characteristics of various patterns in the input by applying multiple dot products (convolutions) to the input matrix. During this stage, an image processing kernel produces a feature map comprising a series of filters. A receptive field, derived from the input, is convolved with a kernel that uses a specific weight set [41]. Typically, after a convolutional layer, a nonlinear operation or function, often referred to as an activation function, follows. The most prevalent activation function is the rectified linear unit (ReLU). Mathematically, the ReLU function and its variants can be expressed as follows:

$f\left( x \right)=max\left( 0,x \right)$ *(1)*

1. **Max Pooling Layer:** This layer down samples the spatial dimensions of the output volume by reducing the number of feature maps and network parameters [42,43].
2. **Dropout Layer:** This layer is utilized as a technique to introduce regularization within the network, ultimately improving generalization. It functions by randomly deactivating certain hidden and visible units, allowing the network to handle diverse internal representations [44].
3. **Batch Normalization:** This normalization adds regularization to the output of the preceding activation layer for each mini-batch by subtracting the batch mean and dividing by the batch standard deviation [39].
4. **Fully Connected Layer:** Also known as the dense layer, this layer produces a single vector based on the input vector's properties. Images are recognized and assigned class labels within these layers. Through back-propagation, the model learns interconnected layers. The number of values in each dense layer determines the model's trainable parameters. The output layer employs the SoftMax activation function, featuring neurons equivalent to the number of classes [45]. The SoftMax function is described by the following formula:

$S(y_{i})=\frac{e^{y_{i}}}{\sum_{j=1}^{m} e^{y_{i}}} for i=1 ............ m$ *(2)*

1. **Flatten Layer**: Dense layers require input in a one-dimensional format, whereas convolutional layers can handle tensor data types. The feature map is vectorized within the flatten layer before being fed into the dense layers [40].
